# Vorteile und Ansätze eines Qualitätsmanagementsystems in biomedizinischen Forschungslaboratorien

**DOI:** 10.1007/s00103-023-03797-y

**Published:** 2023-11-20

**Authors:** Sophia Brünschwitz, Janine Kleymann-Hilmes

**Affiliations:** Koordinierungsstelle Qualitätsmanagement, Robert Koch-Institut, Nordufer 20, 13353 Berlin, Deutschland

**Keywords:** Qualitätsmanagement, Laboratorien, Robert Koch-Institut, Replikationskrise, Biomedizinische Forschung, Quality management, Laboratories, Robert Koch Institute, Replication crisis, Biomedical research

## Abstract

Qualität in der biomedizinischen Forschung ist ein viel diskutiertes Thema unter Expertinnen und Experten, Forschungsinstituten und Förderorganisationen. In der wissenschaftlichen und allgemeinen Presse wird häufig von Qualitätsproblemen berichtet (Bsp.: Ergebnisstabilität nach Testwiederholung – „replication crisis“). Qualitätsmanagementsysteme sind weltweit ein anerkanntes und etabliertes Tool, um Qualität zu lenken und zu leiten sowie Qualitätsprobleme zu beheben. Der Begriff Qualitätsmanagement stößt unter Forschenden jedoch immer wieder auf Widerstand: Geringe Ressourcen, zu starke Regulation, Restriktion der Forschung und unnötige Bürokratie sind hier die Gegenargumente.

Der Gedanke eines Qualitätsmanagementsystems für Forschungslabore ist weltweit nichts Neues. Es bestehen verschiedenste Ansätze von Wissenschaftlerinnen und Wissenschaftlern sowie Organisationen, ein Qualitätsmanagementsystem in Forschungslaboren zu etablieren und für sich ein mehrwertbringendes System zu entwickeln. Ihre Erwartungen: eine Optimierung der Forschungsprozesse sowie eine Effektivitäts- und Effizienzsteigerung.

Dieser Bericht fasst Publikationen zum Thema Qualität in der biomedizinischen Forschung zusammen und erläutert Hintergrund und Vorteile von Qualitätsmanagementsystemen in Organisationen, Laboren und in der Forschung. Als Ausblick wird ein aktuelles Projekt des Robert Koch-Instituts vorgestellt. Der Artikel basiert auf einer Literaturrecherche in englischer und deutscher Sprache. Darüber hinaus wurden international und national gültige Leitfäden berücksichtigt.

## Abstract (en)

Quality in biomedical research is a much-discussed topic among experts, research institutes, and funding organizations. Quality issues are frequently reported in the scientific and general press (e.g., stability of study results after test retake – “replication crisis”). Quality management systems are a globally accepted and established tool to guide and manage quality and to address quality problems. However, the notion of quality management is often met with resistance among researchers: low resources, too much regulation, restriction of research, and unnecessary bureaucracy are counter-arguments.

The idea of implementing a quality management system for research laboratories is nothing new worldwide. There are various approaches by scientists and organizations to establish a quality management system in research laboratories and to develop a value-added system for themselves. Their expectations are an optimization of the research processes as well as an increase in effectiveness and efficiency.

This report summarizes publications concerning quality measures and management in biomedical research and explains the background and advantages of quality management systems in organizations, laboratories, and research. A currently ongoing project of the Robert Koch Institute is presented as an outlook. The article is based on a literature review in English and German. In addition, internationally and nationally applicable and relevant guidelines were considered.

## Einleitung

Die Einhaltung von Standards sind mit dem Ziel der Patientensicherheit in medizinischen Laboratorien der Bundesrepublik Deutschland verpflichtend. Die „Richtlinie der Bundesärztekammer zur Qualitätssicherung laboratoriumsmedizinischer Untersuchungen“ (Rili-BÄK; [1]) ist die Minimalanforderung, die in diesem Rahmen eingehalten werden muss. Darüber hinaus gibt es verschiedene Normen, nach denen Labore akkreditiert werden können. Federführend ist hierbei die DIN EN ISO/IEC 17025 – Allgemeine Anforderungen an die Kompetenz von Prüf- und Kalibrierlaboratorien [2].

Spezifisch für medizinische Laboratorien ist die DIN EN ISO 15189 – Medizinische Laboratorien – Anforderungen an die Qualität und Kompetenz fakultativ zu beachten [3]. Durch die Einhaltung der Normen und Überprüfung durch eine unabhängige externe Akkreditierungsstelle (in Deutschland die Deutsche Akkreditierungsstelle GmbH, DAkkS) belegen Laboratorien ihre Kompetenz national und international. Standardisierungen werden aber mittlerweile auch über die Diagnostiklabore hinaus angestoßen, seien es die mobilen Laboratorien [4] oder Hochsicherheitslaboratorien. Hiervon sowie von der Initiierung verschiedener Qualitätsprojekte innerhalb von Forschungslaboratorien wurde bereits im Februar 2022 in der Veröffentlichung „Qualitätssicherung in medizinischen Laboratorien – Eine Unentbehrlichkeit mit Nutzen und Risiken“ im Bundesgesundheitsblatt [5] berichtet.

In der biomedizinischen Forschung sind anhaltende Qualitätsprobleme bekannt. Der Zeit- und Karrieredruck, Publikationen zu veröffentlichen, ist groß, denn hiervon hängen die Zukunft der Forschenden und Drittmitteleinwerbungen ab. Publikationen werden selten wiederholt und deren Ergebnisse auf Präzision, Genauigkeit und Reproduzierbarkeit geprüft. Die Aussage „publish or perish“ ist allgegenwärtig.

Dieser Bericht basiert auf einer Literaturrecherche, unter Verwendung der Datenbank PubMed und offizieller Websites von Unternehmen, Instituten, Förder- und Standardisierungsorganisationen in englischer und deutscher Sprache. Darüber hinaus wurden international und national gültige Leitfäden und Rechtsnormen berücksichtigt. Es werden verschiedenste Publikationen zum Thema Qualität in der biomedizinischen Forschung zusammengefasst und der Hintergrund und die Vorteile von Qualitätsmanagementsystemen (QMS) in Organisationen, Laboren und in der Forschung erläutert. Als Ausblick wird ein aktuelles Projekt des Robert Koch-Instituts (RKI) zu der Thematik vorgestellt.

## Widerrufe in der Forschung

Impfung löst Autismus aus – eine inhaltlich falsche Publikation mit weitreichenden Folgen. 1998 veröffentlichte Andrew Wakefield, ein britischer Arzt, in der Zeitschrift *The Lancet* einen Bericht über Impfschäden [6]. Er verband Kinderimpfungen mit Autismus. Ein Jahr später wurde dies widerlegt [7]. Aber ein bleibender Schaden war bereits geschehen: Das Vertrauen in Impfungen war gesunken. Auch heute gibt es in weiten Kreisen der Bevölkerung und auch unter Fachleuten diesbezüglich immer noch Bedenken.

Dies ist nur ein Beispiel unter vielen. Die Retraktionszahlen sind seit dem Jahr 2000 angestiegen. Van Noorden berichtet in der Zeitschrift *Nature* von ca. 30 Widerrufen jährlich in den frühen 2000ern und obwohl die Zahl der Veröffentlichungen bis 2011 nur um 44 % gestiegen war, erhöhten sich die Rückrufe um mehr als 1300 % (2011: 400 Rückrufe; [8]).

Besonders eindrücklich zeigt sich dieser Trend auch noch 10 Jahre später in Zusammenhang mit der Widerrufsrate von Veröffentlichungen zu SARS-CoV‑2. Rund ein halbes Jahr nach Beginn der COVID-19-Pandemie im Juni 2020 wurden bereits 17 Arbeiten zurückgezogen [9]. Bis Ende des Jahres 2021 war diese Zahl auf 200 Arbeiten angestiegen [4]. Zurückgezogene Veröffentlichungen im Zusammenhang mit der Pandemie waren nur ein kleiner Anteil aller Widerrufe. Allein im Jahr 2021 wurden insgesamt 3000 Publikationen zurückgenommen [10].

Auch wenn die Zahlen einen klaren Trend spiegeln, sind sie mit Vorsicht zu betrachten, denn steigende Widerrufsraten sind nicht proportional zu steigenden Fehlverhaltensraten. Es gibt verschiedene Gründe für die zunehmende Zahl an Widerrufen, u. a. ein gesteigertes Bewusstsein für wissenschaftliches Fehlverhalten und weiterentwickelte technische Unterstützung, wie Plagiatsfinderprogramme [8]. Auch geben die Widerrufszahlen nicht die reale Anzahl von mangelhaften Veröffentlichungen wieder, es ist von Dunkelziffern auszugehen. Journals gehen sehr unterschiedlich mit Widerrufen um, sei es über die Hintergründe des Widerrufes zu berichten oder überhaupt einen Widerruf zu vollziehen [8]. Dennoch zeigen diese Entwicklungen eine Richtung auf, die Forschende zum Nachdenken anregen sollte.

Ein Widerruf kann mehrere Ursachen haben. Retraction Watch Database berichtet, dass etwa 40 % aufgrund von Fehlern und Problemen mit der Reproduzierbarkeit der Daten vollzogen werden. Der übrige Anteil von Widerrufen erfolgt aufgrund von wissenschaftlichem oder ethischem Fehlverhalten, aber auch Betrug, gefälschte Autorenschaft oder beeinflusste Peer-Reviews sind in diesem Zusammenhang zu nennen [11].

## Reproduzierbarkeit und Replizierbarkeit

Bereits die allgemeine Wochenpresse machte auf die Problematik der Reproduzierbarkeit in der Forschung aufmerksam. Mit der Titelgeschichte „How science goes wrong“ von 2013 warf *The Economist* Wissenschaftlerinnen und Wissenschaftlern vor, zu viel zu vertrauen und zu wenig zu prüfen. In dem Artikel wird von klinischen Studien und einer erheblichen Anzahl involvierter Probanden berichtet, die auf Veröffentlichungen beruhen, die später wegen Fehlern oder Fehlverhalten zurückgezogen wurden [12].

Eine Umfrage der Zeitschrift *Nature* aus dem Jahr 2016 ergab, dass 70 % der teilnehmenden Wissenschaftlerinnen und Wissenschaftler bereits gescheitert waren, ein veröffentlichtes Experiment zu reproduzieren. Darüber hinaus gelang es mehr als der Hälfte nicht, ihr eigenes Experiment zu wiederholen. 52 % der befragten Forschenden erkannten eine „Replikationskrise“, lediglich 3 % sahen diese nicht [13]. Auch wenn diese Umfrage nicht repräsentativ für die globale Wissenschaftsgemeinschaft ist, spiegelt sie eine aktuelle Thematik wider. [14].

Bemerkenswert ist, dass die Begriffe „Reproduzierbarkeit“ und „Replizierbarkeit“ keine einheitliche Definition innehaben ([15], s. Infobox 1). Je nach Wissenschaftsgemeinde kann Reproduzierbarkeit sowohl die erneute Berechnung des Ergebnisses mit denselben Daten [16, S. 46] als auch eine erneute Durchführung des Experiments durch einen dritten Forschenden mit gleichen Ergebnissen [17, S. 9] bedeuten. Letzteres kann aber auch bereits inhaltlich für die Bezeichnung „Replizierbarkeit“ ausreichen [16, 18, S. 12]. Wenn Methoden und Durchführung des Experiments vom Vorgängerexperiment abweichen, dann wird üblicherweise von „Replizierbarkeit“ gesprochen, wie auch im weiteren Verlauf dieses Artikels [17, S. 9].

Reproduzierbarkeit und Replizierbarkeit in der biomedizinischen Forschung zu erreichen, ist bisweilen herausfordernd. Gründe hierfür sind u. a. eine natürliche Variabilität von biologischen Systemen, die Untersuchung dynamischer und komplexer Vorgänge und die Translation von grundlagenwissenschaftlichen Forschungsergebnissen auf den Menschen.

## Gründe für eine „Replikationskrise“

Die Deutsche Forschungsgemeinschaft (DFG) hat offizielle Empfehlungen für Wissenschaftlerinnen und Wissenschaftler der Medizin und Biomedizin zur Verbesserung der Replizierbarkeit herausgegeben. Darin wird auch beschrieben, welche Faktoren eine „nicht Replizierbarkeit“ fördern [18]:

- Konkurrenz um neue publizierbare Erkenntnisse und neue Ressourcen,
- Publikationen als Leistungsnachweis von Forschenden,
- Originalrecherchen werden von Zeitschriften und Magazinen stärker bevorzugt als Replikationen, die Thesen konsolidieren,
- Forschende stehen meist unter Zeitdruck, u. a. durch Förderungsperioden und Vertragslaufzeiten,
- ethische und rechtliche Restriktionen stehen potenziell Replikationen entgegen, besonders bei Versuchen an Tieren und Menschen,
- vermehrtes Fehlen einer geeigneten Infrastruktur zur Archivierung der gewonnenen Daten.

Diese Faktoren ähneln den Ursachen, die in dem *Nature*-Artikel von 2016 [13] aufgeführt werden. Hier werden weitere Aspekte aufgelistet, die zu nicht replizierbaren Forschungsergebnissen beitragen. Das sind weitgehend die Folgen der oben aufgeführten Rahmenbedingungen:

- selektive Berichterstattung,
- geringe statistische Aussagekraft oder schlechte Analyse,
- keine ausreichende Reproduzierbarkeit im Originallabor,
- unzureichende Aufsicht/Mentoring,
- Methoden und Code nicht verfügbar,
- schlechtes experimentelles Design,
- Rohdaten des Originallabors nicht verfügbar,
- Betrug,
- unzureichende Peer-Reviews.

## Ansätze zur Verbesserung der Situation

Um reproduzierbare Daten zu erhalten, muss nach der DFG an folgenden Punkten angesetzt werden: Verwendung von gut durchdachten etablierten und validierten Modellen und Methoden, statistische Planung vor Beginn eines neuen Versuchs, vollständige Beschreibung und Archivierung von generierten Forschungsdaten und geprüften Materialien, welche auch zur weiteren Forschung zur Verfügung gestellt werden, sowie eine adäquate und reproduzierbare Beschreibung von Versuchen, inklusive Hypothese und statistischer Methodik [18]. Im Rahmen der oben erwähnten *Nature*-Umfrage [13] wurden mehr als 1000 Personen gebeten, verschiedene Ansätze zur Verbesserung der Reproduzierbarkeit in der Wissenschaft zu bewerten. Die am häufigsten gewählten Antworten waren „robusteres experimentelles Design“, „bessere Statistiken“ und „bessere Betreuung“.

Die DFG empfiehlt allen Akteuren in der Wissenschaft, tätig zu werden, um die Replizierbarkeit zu erhöhen. Beispielsweise sollten die wissenschaftlichen Einrichtungen ihren Forschenden die notwendigen Ressourcen für ein angemessenes Management ihrer Forschung zur Verfügung stellen. Verlage und Zeitschriften sollten auch verstärkt reproduzierte Ergebnisse und Null- oder negative Ergebnisse veröffentlichen. Darüber hinaus sollten Wissenschaftlerinnen und Wissenschaftler davon absehen, ihre generierten Forschungsdaten zu beschönigen [18].

Die Academy of Medical Sciences (unabhängige britische Organisation für Forschungsförderung in der Biomedizin und Gesundheitsforschung) spricht von einem notwendigen Kulturwandel in der wissenschaftlichen Gemeinschaft [17]. Alle Beteiligten sollten offen sein, über diese Herausforderungen zu sprechen. Es wird ein globaler Ansatz vorgeschlagen, um das Problem anzugehen. Das Bewusstsein sollte geschärft werden, Aus- und Weiterbildungsprogramme zu Forschungsmethoden und statistischem Wissen sollten stattfinden. Darüber hinaus empfiehlt die Academy of Medical Sciences Veröffentlichungsrichtlinien, Peer-Reviews nach der Veröffentlichung und die Vorregistrierung von Protokollen und Analyseplänen.

Viele Ansatzpunkte, die an Forschungsprozesse im einzelnen Labor ansetzen, können sehr gut systematisch und methodisch innerhalb eines QMS koordiniert, umgesetzt und gelenkt werden. Bei stetig steigendem Wettbewerb und Druck auf die Wissenschaftlerinnen und Wissenschaftler aufgrund von Einwerbung von Drittmitteln und Förderperioden unterstützt ein QMS dabei, die Forschungsprozesse zu optimieren und Effektivität und Effizienz zu steigern.

## Qualitätsmanagement in Organisationen

Werden Reproduzierbarkeit und Replizierbarkeit als Qualitätsindikatoren für die biomedizinische Forschung betrachtet, so kann von Qualitätsproblemen gesprochen werden. In Fällen mangelnder Qualität implementieren zumeist kundenorientierte Branchen und Institutionen ein System, das in der Lage ist, ihre Qualität zu lenken und zu leiten. Oft wird ein QMS aufgrund gesetzlicher Anforderungen, wirtschaftlicher Gründe und des Wettbewerbs oder als Reaktion auf ein kritisches Ereignis etabliert.

Innerhalb eines QMS werden angestrebte Ziele definiert, Maßnahmen zur Erreichung dieser festgelegt („plan“) und umgesetzt („do“). Die Maßnahmen werden daraufhin überwacht, ob das gewünschte Ziel erreicht wird („check“). Falls nicht, werden Korrekturmaßnahmen eingeleitet („act“). Dieser kontinuierliche Verbesserungszyklus (PDCA-Zyklus [19]) ist die Basis eines jeden QMS. Dieses Grundprinzip wäre auch für Forschungseinrichtungen anwendbar.

Eine Organisation kann von einem QMS in mehrfacher Hinsicht profitieren: Es verbessert im Idealfall die Betriebsorganisation durch Optimierung der Arbeitsabläufe. Es erleichtert das Erkennen von ineffektiven Prozessen und doppelten Bearbeitungsschritten – das spart Personal, Zeit, Material und Geräte. Darüber hinaus werden Kontrolllücken in Prozessen gefunden und geschlossen, was zu einer Reduzierung der Fehlerkosten führt. Prozesssicherheit ist gegeben, da klare Verantwortlichkeiten und aktuelle Betriebsanweisungen für Methoden, Techniken und Geräte vorhanden sind. Transparenz und Nachvollziehbarkeit sind das Ergebnis eines implementierten QMS.

Des Weiteren fördert es die Erfüllung von Kundenanforderungen (für Forschungslabore sind dies bspw. die Einhaltung der guten wissenschaftlichen Praxis (GWP), vertraglicher Regelungen, der Gesetze sowie ethischer Richtlinien, die Erstellung qualitativ hochwertiger Publikationen, die erfolgreiche Einwerbung von Fördermitteln und Fortführung von Projektförderungen) und erhöht damit den Wettbewerbsvorteil erheblich. Ein QMS führt zu einer spürbaren Risikominderung, u. a. sichert es die Einarbeitung neuer Teammitglieder, die regelmäßigen Schulungen und den Wissenserhalt von ausscheidenden Teammitgliedern.

QMS finden sich in allen Wirtschafts‑, Dienstleistungs‑, Gesundheits‑, Bildungs- und Behördenbereichen wieder, z. B. in der Automobil- [20] und Pharmaindustrie [21], in Krankenhäusern und Universitätskliniken [22], Universitäten [23], Rettungsdiensten und Hilfsorganisationen [24] oder auch in der öffentlichen Verwaltung [25].

Die Erfüllung von Kundenanforderungen ist ein Grundprinzip für den Erfolg. Natürlich folgen unterschiedliche Bereiche verschiedenen Qualitätsstandards, z. B.:

- DIN EN ISO 9001 – Qualitätsmanagementsysteme – Anforderungen [19],
- IATF 16949 – Grundlegende Anforderungen an ein QMS der Automobilindustrie [26],
- KTQ®-Modell – Anforderungskatalog für Gesundheitseinrichtungen in Deutschland und Österreich [27],
- DIN EN 9100:2018 – Qualitätsmanagementsysteme – Anforderungen an Luft‑, Raumfahrt- und Verteidigungsorganisationen [28],
- DIN EN ISO 13485:2021 – Medizinprodukte – Qualitätsmanagementsysteme – Anforderungen für regulatorische Zwecke [29].

Dementsprechend existieren QMS in verschiedenen Formen und Dimensionen, so gestalt- und skalierbar, dass sie den jeweiligen Bedürfnissen der Organisation entsprechen (s. Infobox 2).

Damit ein QMS gelebt wird und den Bedürfnissen der jeweiligen Organisationen und der Stakeholder angepasst bleibt, wird es regelmäßig auf Eignung überprüft. Dies geschieht u. a. durch interne als auch externe Audits. Diese werden von unparteilichen und objektiven Expertinnen und Experten durchgeführt (Bsp.: Akkreditierung durch die DAkkS oder ärztliches Peer Review nach dem Curriculum der Bundesärztekammer als Alternative zum internen Audit [1, 30]).

Um Qualität objektiv nach außen zu belegen, werden Zertifizierungen (Konformitätsbestätigung von Produkten, Prozessen, Dienstleistungen, System oder Personen, Bsp.: DIN EN ISO 9001) oder Akkreditierungen (Kompetenzbestätigung einer Konformitätsbewertungsstelle, Bsp.: DIN EN ISO 15189 und DIN EN ISO/IEC 17025) durchgeführt und eine Zertifizierungs‑/Akkreditierungsurkunde ausgestellt sowie in den jeweiligen Datenbanken veröffentlicht.

## Ein Qualitätsmanagementsystem für Laboratorien

Die Einrichtung eines Qualitätssicherungssystems ist für diagnostische Labore in Deutschland nach § 9 Medizinprodukte-Betreiberverordnung (MPBetreibV) verpflichtend [5, 31]. Eine Grundlage bildet die Rili-BÄK [1]. Darüber hinaus gibt es internationale Standards, die in Deutschland angewendet werden können. ISO 15189 [3] für medizinische Laboratorien und ISO/IEC 17025 [2] für Prüf- und Kalibrierlaboratorien sind bekannte, geprüfte und international akzeptierte Normen, die einer regelmäßigen Revision unterliegen. Hiermit wird sichergestellt, dass die Standards auf dem aktuellen Stand von Wissenschaft und Technik sind.

Medizin- und Prüflaboratorien nutzen und schätzen die Chancen eines QMS ebenso wie die anderen Wirtschaftszweige. Zusätzlich zu den oben genannten Vorteilen profitieren sie von [32]:

- Einhaltung von Vorgaben zur Richtigkeit, Präzision und Genauigkeit von Messwerten,
- Vergleichbarkeit von Messwerten zwischen den Laboren durch Standardisierung und Ringversuche,
- einem präzisen und nachvollziehbaren Weg klinischer Proben vom Eingang bis zum Befund,
- einer präzisen und zielgerichteten Dokumentation,
- verifizierten oder validierten Methoden und Assays,
- regelmäßig gewarteten und kalibrierten Geräten,
- einer Rückführbarkeit der Messungen auf nationale Standards,
- Verfahren für eine genaue Aufzeichnung und Berichterstattung,
- weniger Fehlern und einfacheren Fehlerbehebungen,
- einer Steigerung der Vertrauenswürdigkeit gegenüber Kolleginnen und Kollegen sowie Partnerinnen und Partnern.

Nicht nur stationäre Labore besitzen ein QMS, sondern zukünftig auch Rapid Response Mobile Laboratories (RRML). Eine Standardisierung der RRML findet seit 2017 in einer Zusammenarbeit des Global Outbreak Alert and Response Network (GOARN) und des Regionalbüros der Weltgesundheitsorganisation (WHO) für Europa statt. Seit 2021 sind RRML klassifiziert. Mindeststandards werden derzeit entwickelt [4]. In der Vergangenheit hatte jede Institution ihr eigenes mobiles Labor, das ihren Bedürfnissen und Prioritäten entsprach. Die Standardisierung führt zu einer höheren Interoperabilität der RRML und internationaler Zusammenarbeit auf diesem Gebiet zwischen verschiedenen Ländern. Es soll die schnelle Reaktionsfähigkeit auf einen Ausbruch optimieren [33].

## Ein Qualitätsmanagement für Forschungslabore – Verschiedene Ansätze

Die beschriebenen Vorteile eines QMS bieten auch eine Chance für biomedizinische Forschungslabore: QMS führen zu erhöhter Prozesssicherheit, einer ständigen Verbesserung, Minimierung von Verschwendungen und bieten erhöhte Ergebnisqualität. Dennoch ist ein QMS in der Forschung bisher eine Rarität.

Ein gemeinsamer anerkannter Standard für die Forschung wird in Deutschland durch den Kodex „Leitlinien zur Sicherung der GWP“ der DFG abgebildet [34]. Der heutige Kodex wurde erstmals 1998 als Denkschrift mit der Absicht veröffentlicht, mehr Selbstkontrolle in der Forschung zu erzeugen. Die neueste Ausgabe erschien 2019 mit 19 Leitlinien für wissenschaftliches Arbeiten. Sie sollen eine Kultur der wissenschaftlichen Integrität an Forschungseinrichtungen etablieren. Sie sind verpflichtend einzuhalten, um eine DFG-Förderung zu erhalten [35].

Der Kodex beschreibt sowohl die geltenden Prinzipien (Berufsethos, Verantwortungen, Ombudsperson etc.) als auch Leitlinien für den Forschungsprozess (Qualitätssicherung, Rahmenbedingungen, Forschungsdesign, Dokumentation, Autorenschaft, Archivierung etc.) sowie Verfahren bei Nichtbeachtung. Um die Richtlinien der GWP zu erfüllen, müssen Forschende kein QMS aufbauen. Vielmehr gelten sie als eine Richtschnur für eine vertrauenswürdige Wissenschaft.

Weltweit gibt es verschiedene Ansätze, die Qualität von Forschungsprozessen besser zu managen. Hierfür kommen verschiedene Standards, Normen und Systeme für Forschungslabore infrage.

Einige Institutionen sowie Wissenschaftlerinnen und Wissenschaftler haben begonnen, ein QMS basierend auf der GWP, internationalen und selbst auferlegten Standards zu implementieren, um ihre Forschungsprozesse zu optimieren, Effizienz zu steigern und Wissen bei einer hohen Fluktuation von Mitarbeitenden zu erhalten. In Deutschland sind das z. B. die Bundesanstalt für Materialforschung und -prüfung (BAM) als Ressortforschungseinrichtung sowie das Berlin Institute of Health an der Charité (BIH).

Das BIH entwickelte zwei QMS, um die Qualität präklinischer Forschungsdaten zu verbessern:

- EQIPD (Enhancing Quality in Preclinical Data, ursprünglich European Quality in Preclinical Data; [36]),
- PREMIER (Predictiveness and Robustness through Modular Improvement of Experimental Research; [37]).

Gefördert von der Innovative Medicines Initiative (IMI) der Europäischen Union wird EQIPD seit 2017 stetig weiterentwickelt. Gründungsmitglieder sind 29 Institutionen aus 8 verschiedenen Ländern. Es soll die frühe Arzneimittelforschung strukturierter verlaufen lassen [38] und in jeder Art Organisation von einer großen Universität, einem Forschungsinstitut oder Unternehmen bis hin zu einem einzelnen Labor implementiert werden können. EQIPD basiert auf 18 Kernanforderungen, die erfüllt werden müssen [39]. Eine Zertifizierung des EQIPD-Systems ist möglich. Bis März 2022 haben 6 Forschungseinrichtungen ein solches Zertifikat erhalten; darunter die Deutsche Mausklinik (Institut für Experimentelle Genetik, Helmholtz Zentrum München) oder der Forschungsbereich Präklinische Forschung, In-vivo-Pharmakologie, Fraunhofer-Institut für Translationale Medizin und Pharmakologie ITMP, Industriepark Höchst [40].

PREMIER ist ein Rahmenkonzept eines modularen QMS mit Mindestanforderungen, das an die individuellen Bedürfnisse des Labors angepasst werden kann. Das Konzept besteht seit 2017 und wird durch die VW-Stiftung gefördert. Es handelt sich um ein skalierbares System, d. h., es ist dem Labor freigestellt, ob das gesamte PREMIER-System implementiert wird oder nur ein Teil der Module [41].

EQIPD und PREMIER sollen flexibel und benutzerfreundlich sein und auf die individuellen Bedürfnisse eingehen, ohne den Forschergeist einzuschränken.

Das BIH hat eigene Managementsysteme geschaffen, da die Anwendbarkeit der ISO 9001 – „Qualitätsmanagementsysteme – Anforderungen“ [19], die 2014 in der Abteilung für experimentelle Neurologie an der Charité Universitätsmedizin Berlin eingeführt wurde, nicht zufriedenstellend war. Sie wurde als zu starr angesehen und durch die allgemeine Anwendbarkeit innerhalb verschiedenster Organisations- und Unternehmensarten als fachfremd und nicht passend wahrgenommen. Jedoch wurden auch Verbesserungen durch die Implementierung von ISO 9001 festgestellt, wie eine bessere Fehlerkultur und Verantwortlichkeitsverteilung und ein optimierter Umgang mit Ressourcen und Wissen sowie verbesserte Protokollierungen [42].

Diese Vorteile konstatierte auch das Labor für Molekularbiologie und Funktionelle Genomik der Technischen Hochschule Wildau im Jahr 2010. Sie führten ein QMS nach ISO 9001 ein und berücksichtigten zusätzlich ISO/IEC 17025. Sie berichteten von verbesserten Prozessen und einem Anstieg in der Ergebnisqualität bei gleichzeitiger Ressourcenschonung [43].

Weitere Forschungseinrichtungen haben bereits ihre Erfahrungen und Ansätze mit einem QMS veröffentlicht. So berichtet z. B. die Heinrich Heine Universität Düsseldorf [44] über ein QMS in der Forschung, welches sich v. a. durch Erschwinglichkeit und schnelle Einführung herausheben soll. Es basiert auf klassischen QMS-Ansätzen, wie Arbeitsanweisungen und Labormeetings, sowie auf einem elektronischen Laborbuch, Automatisierungen und einer Biobank.

Basierend auf der UNE 166002, ISO/IEC 17025 und agilen Prinzipien hat die Polytechnische Universität Madrid in Zusammenarbeit mit dem spanischen RedLab-Netzwerk Anforderungen für Forschungslabore erstellt. In einer Umfrage innerhalb des RedLab-Netzwerkes wurden u. a. folgende Punkte als Vorteile eines QMS in der Forschung identifiziert: vereinfachte und optimierte Abläufe, Wissensmanagement und Qualitätsverbesserung der Datenerhebung und Archivierung [45].

Obwohl bereits über positive Erfahrungen mit QMS in Forschungslaboren berichtet wurde, gibt es viele Gegensprecher dieser Strategie. Angeführte Argumente sind: Ein QMS ist zu starr für die Komplexität von Forschung und steigert die Bürokratie. Die Kreativität und der Forschergeist werden eingeschränkt. Ressourcen sind begrenzt, bestehende QMS sind für die Forschung nicht geeignet und für den Studienerfolg nicht notwendig. Forschung funktionierte schon immer ohne QMS und so auch in Zukunft [42, 45]. Dies sind zweifellos Einwände, die vor Einführungen eines QMS geprüft werden sollten, aber ein QMS nicht generell ausschließen.

Gute Forschungsansätze und der „bahnbrechende Gedanke“ entstehen in der Regel nicht durch ein QMS. Das Qualitätsmanagement kann aber bei der Umsetzung von wissenschaftlichen Fragestellungen wertvoll sein und Nutzen für alle Beteiligten – und damit für die Gesellschaft – stiften. Ein QMS soll Wissenschaftlerinnen und Wissenschaftler bei ihrer Forschung unterstützen und Effektivität und Effizienz steigern. Des Weiteren wird der Forschungsprozess strukturiert und optimiert. Dies trägt dazu bei, die Verschwendung von Ressourcen und Zeit zu reduzieren. Das Personal wird regelmäßig und bedarfsorientiert geschult, Verantwortlichkeiten sind klar geregelt, Wissen bleibt erhalten. Darüber hinaus werden eine geordnete, sichere Datenspeicherung und eine positive Fehlerkultur gefördert sowie Risiken reduziert [42]. Um das zu erreichen, kann ein QMS gut unterstützen, gerade bei steigender Komplexität der Forschungsprojekte.

## Ausblick

Mittels eines QMS werden viele Schwachstellen in Forschungsprozessen beherrschbar. Ein Beispiel hierfür sind eine verbesserte und gezielte Einarbeitung und Mentoring von jungen Forschenden durch ein standardisiertes Vorgehen. Viele befürchten aber auch eine Einschränkung der Forschungsfreiheit und Verschwendung von Ressourcen durch die Einführung eines QMS. Um diese Problematik zu klären, wird eine Forschung an Forschungsprozessen benötigt. Das wurde auch bereits von der Academy of Medical Sciences erkannt [17, S. 64].

Aufgrund der Aktualität fördert das RKI als zentrale staatliche wissenschaftliche Einrichtung auf dem Gebiet der biomedizinischen Forschung und nationales Public-Health-Institut ein internes Projekt zur Implementierung und Validierung eines QMS für seine Forschungslabore. Damit erweitert es sein bestehendes QMS und formt eine einheitliche Infrastruktur [34].

Als erster Schritt werden Anforderungen an die biomedizinischen Forschungslabore definiert. Das geschieht u. a. in Zusammenarbeit mit den Forschungslaboratorien des RKI sowie mit dem Fachgebiet „Angewandte und Molekulare Mikrobiologie“ der Fakultät III – Prozesswissenschaften der Technischen Universität Berlin und der DAkkS. Die Anforderungen werden unter den Forschenden diskutiert und in den Laboren etabliert. Sie müssen folgenden Ansprüchen genügen:

- GWP als Grundlage,
- keine Beeinträchtigung des Forschergeistes,
- Unterstützung der täglichen Laborarbeit,
- Schaffung evidenzbasierter Maßnahmen,
- keine unverhältnismäßige Ressourcenbindung.

Es soll eine Validierung der Anforderungen stattfinden, um Aussagen über Wirksamkeit und Mehrwert der Maßnahmen treffen zu können. Hierfür werden Knowledge-Attitude-Practices-(KAP-)Befragungen mit dem Laborpersonal durchgeführt, um ihr Wissen, ihre Einstellung und Praktiken zu erfassen. Des Weiteren werden Kennzahlen für Forschungslabore erhoben. Die Kennzahlen sollen messen, ob sich das QMS etabliert, langfristig aufrechterhalten und weiterentwickelt wird und es einen Mehrwert bildet. Interne Peer-Reviews dienen als Implementierungs- und Wirksamkeitskontrolle.

Durch eine Auswertung der erhaltenen Daten können Rückschlüsse auf Praktikabilität und Wirkung der Forschungsstandards erfolgen.

## Fazit

Bei stetig steigendem Wettbewerb und Druck in der Wissenschaft aufgrund von Drittmitteleinwerbung und Förderperioden bildet ein QMS eine Basis, die Forschungsprozesse optimiert und Effektivität und Effizienz steigert. Die Vorteile eines QMS können auch für Forschungslabore eine Chance bieten: kontinuierliche Verbesserung, Planung von Ressourcen, Einsparung von Zeit und Kosten, Reduktion von Risiken, Klärung von Verantwortlichkeiten und Wissenserhalt ausscheidender Teammitglieder. Im Gegensatz zu medizinischen Laboren besteht ein QMS in der Forschung auf freiwilliger Basis. Es ist die Entscheidung einer jeden Organisation, ob einer solchen Strategie eine Chance gegeben wird, um die Forschungsqualität seiner Wissenschaftlerinnen und Wissenschaftler zu sichern und nach außen transparent zu machen. Ansätze für ein QMS in der Forschung gibt es jedenfalls vielfältige.

### Infobox 1 Definitionen von Replizierbarkeit und Reproduzierbarkeit

- National Academies of Sciences, Engineering, and Medicine (Dachorganisation der 3 US-amerikanischen Wissenschaftsakademien, welche die US-Politik in wissenschaftlichen Fragen unabhängig und objektiv berät) [16, S. 46], nota bene: übersetzt aus dem englischen Original:

„Reproduzierbarkeit bedeutet, mit denselben Eingabedaten konsistente Ergebnisse zu erzielen; Berechnungsschritte, Methoden und Code; und Analysebedingungen. Diese Definition ist gleichbedeutend mit *rechnerischer Reproduzierbarkeit* …“

„Replizierbarkeit bedeutet, konsistente Ergebnisse über Studien hinweg zu erhalten, die darauf abzielen, dieselbe wissenschaftliche Frage zu beantworten, von denen jede ihre eigenen Daten erhalten hat.“

- Academy of Medical Sciences (unabhängige britische Organisation für Forschungsförderung in der Biomedizin und Gesundheitsforschung), Biotechnology and Biological Sciences Research Council (BBSRC, Förderorganisation für nicht-medizinische Biowissenschaften), Medical Research Council (MRC, britische Forschungsorganisation für den Bereich Medizin und verwandte Fachgebiete) und Wellcome Trust (gemeinnützige Treuhand für Forschungsförderung) [17, S. 9], nota bene: übersetzt aus dem englischen Original:

„Ergebnisse sind reproduzierbar, wenn ein unabhängiger Forscher ein Experiment unter ähnlichen Bedingungen wie bei einer früheren Studie durchführt und entsprechende Ergebnisse erzielt.“

„Eine Replikationsstudie dient dazu, die Reproduzierbarkeit zu testen. Obwohl sie der Originalstudie ähnlich sein sollte, muss eine Replikationsstudie methodisch nicht identisch sein. … Das Hauptziel besteht darin, festzustellen, dass die ursprünglichen Ergebnisse von einem unabhängigen Forscher mit ähnlichen Methoden und Analysen wiederholt werden können.“

- Deutsche Forschungsgemeinschaft (DFG) [18, S. 12]:

„Die Begriffsbildung der Wörter Replikation, Replizierbarkeit bzw. der ebenfalls oft verwendeten Begriffe Reproduktion, Reproduzierbarkeit sowie die Abgrenzungen der Bedeutung zwischen diesen Ausdrücken ist noch nicht abgeschlossen. Die Begriffe werden auch im angelsächsischen Sprachraum nicht einheitlich verwendet.“

„Replikation ist die Wiederholung einer Untersuchung/eines Experiments/einer Studie, die den Anspruch erhebt, wiederholbar zu sein, aber auch allgemein die unbegrenzte Möglichkeit bedeutet, etwas zu wiederholen oder ein Experiment noch einmal durchführen zu können. Entsprechend bedeutet Replizierbarkeit die Möglichkeit oder Fähigkeit, Ergebnisse innerhalb des Fehlerrahmens wiederholt zu bestätigen.“

### Infobox 2 Qualitätsmanagementsysteme (QMS)

- ISO 9001 [19] beschreibt die Anforderungen an ein QMS. Die Norm basiert auf dem PDCA-Zyklus („plan, do, check, act“) sowie auf einem risikobasierten Vorgehen. Als prozessorientierte Norm bildet sie ein systematisches Werkzeug, um Anforderungen von Kundinnen und Kunden und interessierten Parteien zu erfüllen. Darüber hinaus schafft die ISO Vertrauen in Produkte und Dienstleistungen. Das Dokument stellt die Grundlage vieler weiterer Richtlinien und Anforderungskataloge dar, wie z. B. für die ISO 15189 für medizinische Laboratorien und ISO/IEC 17025 für Prüf- und Kalibrierlaboratorien.
- Des Weiteren gibt es sogenannte Total-Quality-Management-(TQM-)Systeme, die auf den nachhaltigen Unternehmenserfolg ausgerichtet sind. Sie beziehen anders als QMS u. a. die Beherrschung aller Geschäftsprozesse sowie die Mitarbeiterorientierung mit ein. Auch Themen wie Finanzen werden integriert. Ziel ist die Steigerung der Effektivität und Effizienz des Unternehmens. Ansätze für TQM bilden u. a. der Leitfaden ISO 9004 [46] sowie das European-Foundation-for-Quality-Management-Modell (EFQM; [47]). Ein Grundbaustein der ISO 9004 und des EFQM-Modells ist die Selbstbewertung der eigenen Organisationen, um Stärken und Verbesserungspotenziale zu erkennen und eine Priorisierung von Themenfeldern vorzunehmen. Das EFQM folgt einem ähnlichen Prinzip wie die ISO-Normen. Die RADAR-Logik (R – Results/Ergebnisse, A – Approach/Vorgehen, D – Deployment/Umsetzung, A – Assessment und R – Refinement/Bewertung und Verbesserung) des EFQM-Modells ist vergleichbar mit dem PDCA-Zyklus. Erst werden die gewünschten Ergebnisse definiert, die man als Organisation innerhalb seiner Strategie erreichen möchte. Danach werden die Vorgehensweisen festgelegt, diese werden umgesetzt und schließlich die Maßnahmen bewertet und verbessert, um die gesetzten Ziele zu erreichen. Hierdurch ist ein kontinuierlicher Verbesserungsprozess gewährleistet.

## References

[1] Bundesärztekammer (2023). Richtlinie der Bundesärztekammer zur Qualitätssicherung laboratoriumsmedizinischer Untersuchungen. Deutsches Ärzteblatt p120 (121-122): A-994/B-858.

[2] DIN Deutsches Institut für Normung e. V. (2018) DIN EN ISO/IEC 17025:2018-03. Allgemeine Anforderungen an die Kompetenz von Prüf- und Kalibrierlaboratorien. https://www.beuth.de/de/norm/din-en-iso-iec-17025/278030106. Zugegriffen: 3. Aug. 2022

[3] DIN Deutsches Institut für Normung e. V. (2023) DIN EN ISO 15189:2023-03. Medizinische Laboratorien - Anforderungen an die Qualität und Kompetenz. https://www.beuth.de/de/norm/din-en-iso-15189/362884991. Zugegriffen: 3. Aug. 2023

[4] World Health Organization Regional Office for Europe (2021). Guidance for rapid response mobile laboratory (RRML) classification.

[5] Kleymann-Hilmes J, Brünschwitz S, Müller M (2022). Qualitätssicherung in medizinischen Laboratorien – Eine Unentbehrlichkeit mit Nutzen und Risiken. Bundesgesundheitsblatt Gesundheitsforschung Gesundheitsschutz.

[6] Wakefield AJ, Murch SH, Anthony A (1998). RETRACTED: Ileal-lymphoid-nodular hyperplasia, non-specific colitis, and pervasive developmental disorder in children. The Lancet.

[7] Taylor B, Miller E, Farrington C (1999). Autism and measles, mumps, and rubella vaccine: no epidemiological evidence for a&nbsp;causal association. Lancet.

[8] Van Noorden R (2011). Science publishing: the trouble with retractions. Nature.

[9] Yeo-Teh NSL, Tang BL (2019). An alarming retraction rate for scientific publications on Coronavirus Disease 2019 (COVID-19). Accountability in Research.

[10] Retraction Watch (2021) The top retractions of 2021. The Scientist https://www.the-scientist.com/news-opinion/the-top-retractions-of-2021-69533. Zugegriffen: 28. Febr. 2022

[11] Brainard J (2018). What a massive database of retracted papers reveals about science publishing’s ‘death penalty. Science.

[12] Anonymous (2013) How science goes wrong. The Economist. The Economist Newspaper https://www.economist.com/leaders/2013/10/21/how-science-goes-wrong. Zugegriffen: 28. Febr. 2022

[13] Baker M (2016). 1,500 scientists lift the lid on reproducibility. Nature.

[14] Anonymous (2016). Reality check on reproducibility. Nature.

[15] Goodman SN, Fanelli D, Ioannidis JP (2016). What does research reproducibility mean?. Sci Transl Med.

[16] National Academies of Sciences, Engineering and Medicine (2019). Reproducibility and replicability in science / committee on reproducibility and replicability in science.

[17] Academy of Medical Sciences, Biotechnology and Biological Sciences Research Council, Medical Research Council, Wellcome Trust (2015) Reproducibility and reliability of biomedical research: improving research practice. https://acmedsci.ac.uk/file-download/38189-56531416e2949.pdf. Zugegriffen: 24. Jan. 2022

[18] Forschung SSfGidK (2018) Replizierbarkeit von Ergebnissen in der Medizin und Biomedizin; Stellungnahme der Arbeitsgruppe „Qualität in der Klinischen Forschung“ der DFG-Senatskommission für Grundsatzfragen in der Klinischen Forschung. https://www.dfg.de/download/pdf/dfg_im_profil/geschaeftsstelle/publikationen/stellungnahmen_papiere/2018/180507_stellungnahme_replizierbarkeit_sgkf.pdf. Zugegriffen: 25. Nov. 2022

[19] DIN Deutsches Institut für Normung e. V. (2015) DIN EN ISO 9001:2015–11. Qualitätsmanagementsysteme – Anforderungen. https://www.beuth.de/de/norm/din-en-iso-9001/235671251. Zugegriffen: 3. Aug. 2023

[20] Volkswagen Aktiengesellschaft (2019) Konzern-Qualitätsmanagement. https://geschaeftsbericht2018.volkswagenag.com/konzernlagebericht/nachhaltige-wertsteigerung/konzern-qualitaetsmanagement.html. Zugegriffen: 25. Nov. 2022

[21] Pfizer (2022) https://www.pfizer.com/about/quality#:~:text=Pfizer%E2%80%99s%20Quality%20Policy%20is%20implemented%20through%20a%20comprehensive,of%20quality%20for%20our%20patients%2C%20customers%2C%20and%20stakeholders. Zugegriffen: 25. Nov. 2022

[22] CU Berlin (2023) Klinisches Qualitäts- und Risikomanagement der Charité Berlin. https://qualitaetsmanagement.charite.de/. Zugegriffen: 7. Aug. 2023

[23] TU Berlin (2020) Strategisches Controlling: Gruppe Qualitätsmanagement, Studienreform und Kennzahlen. https://www.tu-berlin.de/qualitaet/. Zugegriffen: 2. Dez. 2022

[24] TÜV Süd (2022) Johanniter in Bayer ISO 9001. https://www.tuvsud.com/de-de/wissenswert/newsletter/value-newsletter/1-2021/johanniter-in-bayern-iso-9001-zertifiziert. Zugegriffen: 2. Dez. 2022

[25] Heimat BdIuf (2022) Qualitätsmanagement. https://www.bmi.bund.de/DE/themen/moderne-verwaltung/verwaltungsmodernisierung/qualitaetsmanagement/qualitaetsmanagement-node.html. Zugegriffen: 2. Dez. 2022

[26] IATF Global Oversight (2023) About. https://www.iatfglobaloversight.org/iatf-169492016/about/. Zugegriffen: 10. Febr. 2022

[27] Gesundheitswesen Das KTQ-Modell. https://ktq.de/fileadmin/media/info/3021307_KTQ-Risikomanagement_a.pdf. Zugegriffen: 10. Febr. 2022

[28] DIN Deutsches Institut für Normung e. V. DIN EN ISO 9100:2018–08. Qualitätsmanagementsysteme – Anforderungen an Organisationen der Luftfahrt, Raumfahrt und Verteidigung. https://www.beuth.de/de/norm/din-en-9100/289865083. Zugegriffen: 2022

[29] DIN Deutsches Institut für Normung e. V. (2021) DIN EN ISO 13485:2021-12. Medizinprodukte – Qualitätsmanagementsysteme – Anforderungen für regulatorische Zwecke. https://www.beuth.de/de/norm/din-en-iso-13485/332674603. Zugegriffen: 3. Aug. 2023

[30] Bundesärztekammer (2013) Curriculum Ärztliches Peer Review. https://www.bundesaerztekammer.de/fileadmin/user_upload/_old-files/downloads/CurrAerztlPeerReview2013.pdf. Zugegriffen: 8. Aug. 2023

[31] Anonymous (2021) Medizinprodukte-Betreiberverordnung in der Fassung der Bekanntmachung vom 21. August 2002 (BGBl. I S. 3396), die zuletzt durch Artikel 7 der Verordnung vom 21. April 2021 (BGBl. I S. 833) geändert worden ist. https://www.gesetze-im-internet.de/mpbetreibv/BJNR176200998.html. Zugegriffen: 20. Sept. 2022

[32] Brünschwitz S, Kleymann-Hilmes J, Mielke M, Schaade L, Wieler L (2020) The quality management system of the Robert Koch institute. 17th IMEKO TC 10 and EUROLAB Virtual Conference “Global Trends in Testing, Diagnostics & Inspection for 2030” p53 - 60. https://www.imeko.info/publications/tc10-2020/IMEKO-TC10-2020-003.pdf. Zugegriffen: 10. Febr. 2022

[33] WHO Europe (2019) New generation of mobile laboratories improve rapid response to health emergencies. https://www.who.int/europe/news/item/19-12-2019-new-generation-of-mobile-laboratories-improve-rapid-response-to-health-emergencies. Zugegriffen: 25. Nov. 2022

[34] Deutsche Forschungsgemeinschaft (2019) Guidelines for safeguarding good research practice. Code of conduct. https://zenodo.org/record/3923602. Zugegriffen: 29. Jan. 2022

[35] Deutsche Forschungsgemeinschaft (2022) Gute wissenschaftliche Praxis. https://www.dfg.de/foerderung/grundlagen_rahmenbedingungen/gwp/index.html. Zugegriffen: 25. Nov. 2022

[36] EQIPD EQIPD. https://quality-preclinical-data.eu/. Zugegriffen: 3. Febr. 2022

[37] PREMIER PREMIER. https://premier-qms.org/. Zugegriffen: 3. Febr. 2022

[38] EQIPD OUR VISION. https://quality-preclinical-data.eu/about-eqipd/our-vision/. Zugegriffen: 15. Febr. 2022

[39] Bespalov A, Bernard R, Gilis A (2021). Introduction to the EQIPD quality system. Elife.

[40] EQIPD (2022) Research units that have successfully implemented the EQIPD quality system and received a certificate already. https://quality-preclinical-data.eu/about-eqipd/eqipd-certified-research-groups/. Zugegriffen: 25. Nov. 2022

[41] PREMIER (2022) The clickable PREMIER “House”. https://premier-qms.org/premier. Zugegriffen: 25. Nov. 2022

[42] Dirnagl U, Kurreck C, Castaños-Vélez E, Bernard R (2018). Quality management for academic laboratories: burden or boon?. EMBO Reports.

[43] Weinert S, Wohlfahrt I, Schmid A, Frohme M (2010). Einführung eines Qualitätsmanagement-Systems in einem molekularbiologischen Labor einer Hochschule.

[44] Hewera M, Nickel AC, Knipprath N (2020). An inexpensive and easy-to-implement approach to a quality management system for an academic research lab. F1000Res.

[45] Martínez-Perales S, Ortiz-Marcos I, Ruiz JJ (2021). A proposal of model for a quality management system in research testing laboratories. Accred Qual Assur.

[46] DIN Deutsches Institut für Normung e. V. (2018) DIN EN ISO 9004:2018-08 – quality management – quality of an organization – guidance to achieve sustained success. https://www.beuth.de/de/norm/din-en-iso-9004/283875061. Zugegriffen: 3. Aug. 2023

[47] EFQM (2022) Organisationen verbessern. https://efqm.org/de/. Zugegriffen: 23. Sept. 2022

